# A Case Study of Multiple Endocrine Neoplasia Type 2A

**DOI:** 10.7759/cureus.27504

**Published:** 2022-07-31

**Authors:** Chim M Yang

**Affiliations:** 1 Otolaryngology - Head and Neck Surgery, Western Reserve Hospital, Cuyahoga Falls, USA

**Keywords:** neck mass, family thyroid cancer, calcitonin, multiple endocrine neoplasia type 2a, medullary thyroid carcinoma

## Abstract

Multiple endocrine neoplasia type 2 is an autosomal dominant neoplastic syndrome with subtypes multiple endocrine neoplasia type 2A, multiple endocrine neoplasia type 2B, and familial medullary thyroid carcinoma. Medullary thyroid carcinoma universally coincides with multiple endocrine neoplasia type 2. Multiple endocrine neoplasia type 2A is a rare disease and the affected patients are generally asymptomatic. The morbidity and mortality are mainly due to medullary thyroid carcinoma and often proper clinical workup is warranted for expedited surgical intervention. Total thyroidectomy along with neck dissection may be required for disease control. This report will cover a patient who presented with medullary thyroid carcinoma and was worked up to have multiple endocrine neoplasia type 2A. She underwent total thyroidectomy with central neck dissection.

## Introduction

Multiple endocrine neoplasia type 2 (MEN2) is a rare neoplastic syndrome inherited in an autosomal dominant fashion consisting of MEN2A, MEN2B, and familial medullary thyroid carcinoma (MTC) [1]. MEN2 is characterized by the 100% prevalence of MTC and an increased risk of developing other specific tumors affecting additional glands of the endocrine system due to the mutations in the RET gene, located on chromosome 10q11.2 [1]. Major characteristics of MEN2A include MTC, pheochromocytoma, and hyperparathyroidism. MEN2B is characterized by MTC, pheochromocytoma, multiple mucosal neuromas, and often a marfanoid habitus [2,3]. This mutation leads to the alterations of C cells derived from the neural crest [1]. In patients with MEN2A syndrome, prophylactic thyroidectomy is recommended due to carriers having a 100% risk of developing MTC during their lifetime [4,5].

MEN2A represents 95% of all MEN2 while MEN2B represents 5% of the remaining cases [6]. MTC leads to fatal outcomes in MEN2A if it is inappropriately treated or goes undiagnosed. In familial cases where the genetic diagnosis is made with RET mutation gene, a prophylactic thyroidectomy significantly reduces morbidity and mortality [7]. Pheochromocytoma is rarely the first manifestation of MEN2A and is often identified during screening exams in patients with MEN2. Pheochromocytoma occurs in 40% of patients with MEN2A and is usually evident about 10 years later after MTC or C cell hyperplasia [3,8].

## Case presentation

**Figure 1:**
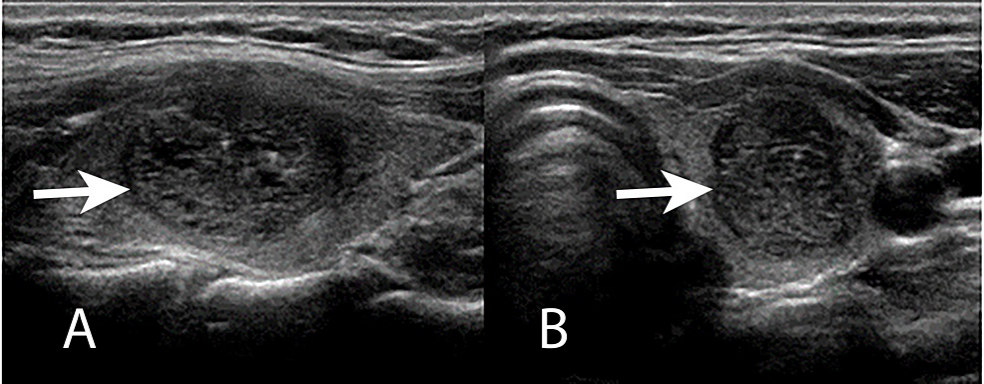
Left thyroid ultrasound (A) Sagittal view. (B) Transverse view. Bold arrows point to the nodule.

A 33-year-old female was referred to the ENT practice by her primary care physician who presented with a left thyroid nodule, difficulty swallowing, and left neck mass. Her vitals were normal and euthyroid with calcium of 8.9 mg/dL (normal value 8.6-10.3 mg/dL). Ultrasound of the thyroid gland shows a 3 cm left thyroid nodule with irregular borders, and intranodular vascularity (Figure 1A, 1B).

Upon further history, she noted thyroid and adrenal disease in her family. She was estranged from her family over personal family issues at a young age, so she was unsure about the specifics regarding the thyroid and adrenal disease. Given the ultrasound findings, a fine-needle aspiration (FNA) showed salt-and-pepper chromatin, multinucleation, and the presence of amyloid, which was consistent with a diagnosis of MTC. Given the FNA result, a laboratory test was obtained. In the laboratory tests, calcitonin was 377 pg/mL (normal value <10 pg/mL) and plasma metanephrines were 13 pg/mL (normal value 12-60 pg/mL). Carcinoembryonic antigen was 87 ng/mL (normal 0-2.5 ng/mL).

Due to the frequent involvement of cervical lymph nodes and multicentricity of disease in the thyroid, the initial surgical management included total thyroidectomy along with bilateral central neck dissection and bilateral level 2-5 neck lymph node dissection. Jackson Pratt drain that was placed into the surgical bed prior to closure was removed when the drain output was less than 30 cc over 24 hours. She was admitted for overnight observation and discharged the following day with a prescription of 125 mg of levothyroxine.

**Figure 2:**
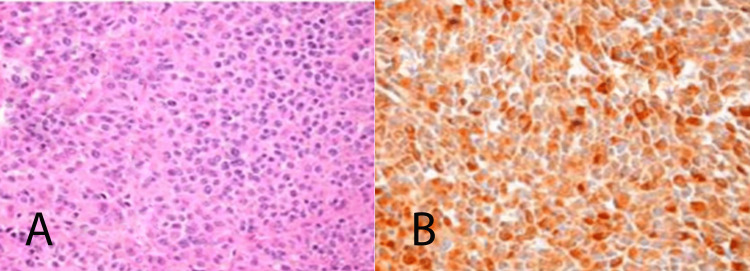
Histopathology Hematoxylin and eosin (A) at 400x magnification. Calcitonin immunohistochemcial stain (B) at 400x magnification.

Histochemical investigation with hematoxylin and eosin shows small round blue cells along with immunohistochemical staining for calcitonin being positive in the tumor (Figure 2A, 2B). These results were consistent with MTC. Medullary carcinoma was also found to be in three lymph nodes removed from the central neck dissection as well as one level III lymph node on the left.

**Figure 3:**
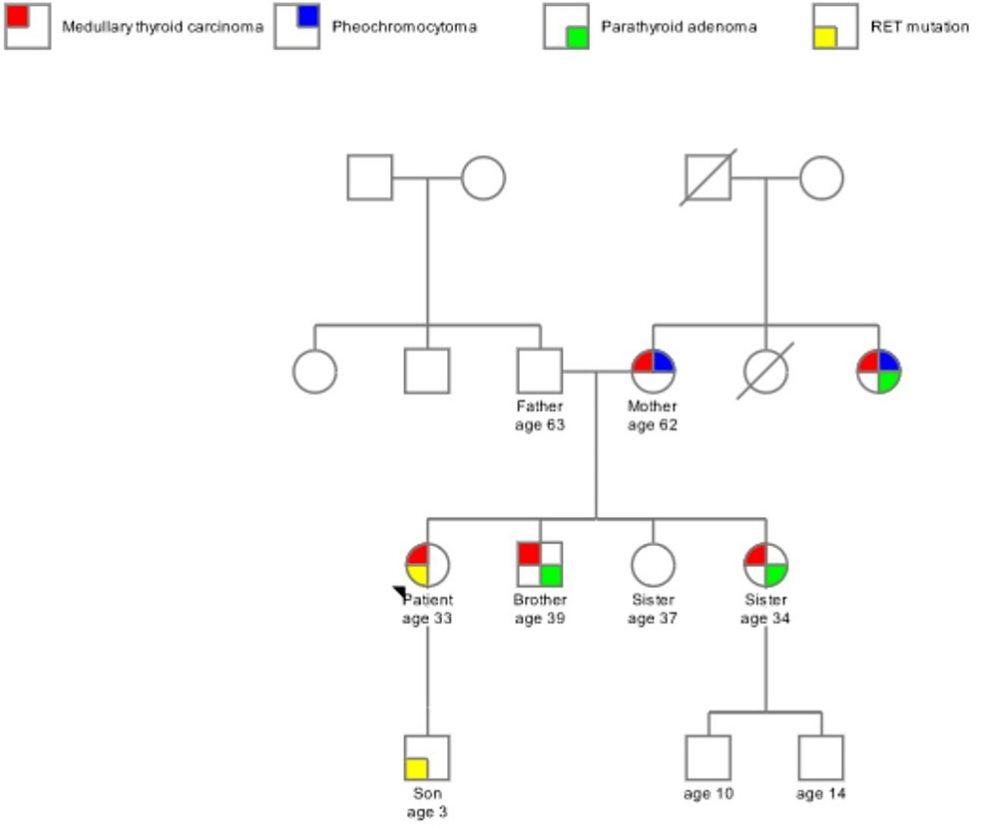
Family pedigree Arrowhead points to the patient (proband). Multigenerational pedigree of the family. RET mutation was only tested on the patient and her son. The rest of the pedigree was obtained from the family history from the patient.

At the four-month postoperative follow-up, calcitonin was 6 pg/mL (normal value <10 pg/mL) and carcinoembryonic antigen was 1 ng/mL (normal 0-2.5 ng/mL), showing great surgical control of her MTC. Genetic testing was carried out with peripheral whole blood for the RET mutation. The genetic analysis was performed for the RET full gene analysis which utilizes sequencing to detect single nucleotide and copy number variants in one gene associated with MEN2. Her results were positive for the V804M RET germline proto-oncogene mutation which aids to confirm her diagnosis of MEN2A. She has a three-year-old son and genetic testing was also positive for V804M RET germline proto-oncogene mutation. The son was referred to a children’s hospital for further evaluation and management. On her postoperative visit and learning of her diagnosis, she was able to reach out to her family to obtain a family history. The genetic pedigree was suggestive of MEN2A given the syndrome in her other family member consisting of MTC, parathyroid adenoma, and pheochromocytoma (Figure 3). The family members have since reached out to obtain their genetic tests.

## Discussion

MEN2 is a rare hereditary disease characterized by the presence of MTC, unilateral or bilateral pheochromocytoma, and primary parathyroid hyperplasia [2]. The lifetime penetrance of MTC is nearly 100%, and there is variability in the other manifestations [6]. Patients should be properly worked up when one of the syndromes is identified for MEN2 and family history should also be taken into account.

In an MEN2 patient, testing should be performed for immediate family members specific to the RET mutation [1]. Genotyping should be done before the time of prophylactic thyroidectomy. The risk of developing pheochromocytoma is variable with regard to penetrance [9]. Follow-up routine surveillance every year with serum metanephrines or 24-hour urinary metanephrine is required [10]. Hyperparathyroidism in MEN2A is often mild and asymptomatic and yearly screening should be performed with serum calcium. If calcium levels are elevated, an intact parathyroid hormone is indicated [11].

The American Thyroid Association guidelines recommend that prophylactic lateral neck dissections may be considered based on serum calcitonin levels. Prophylactic ipsilateral central and lateral neck dissection should be considered for patients with basal serum calcitonin 20 pg/mL, and contralateral lateral neck dissection should be considered for serum calcitonin >200 pg/mL [4,5]. 

A study done by Mathiesen et al. found that mortality in MEN2 is mainly due to MTC. The 10-year disease-specific survival rate was 98%, 93%, 87%, and 53% for MTC stages I, II, III, and IV, respectively, and the overall 10-year survival is estimated to be 64% [12]. Pheochromocytomas are a rare cause of death in MEN2 patients [9]. The strongest prognostic factor is the stage of the disease and the presence of extrathyroidal metastases at presentation [12,13]. Postoperative calcitonin within the normal range is associated with a 10-year survival rate of 98% while calcitonin doubling times were associated with aggressive disease progression and decreased overall survival [12,14].

## Conclusions

MEN2 is a rare disease and the affected patients are generally asymptomatic. This case study illustrates the importance of recognizing the clinical manifestations of MEN2. Early diagnosis and optimal workup allow for prompt interventions and to decrease the morbidity and mortality associated with MEN2. Affected families should be managed with RET genetic testing and follow-up regarding the diagnosis. Mortality of MEN2 is due to MTC and prophylactic total thyroidectomy is warranted. Patients will require long-term follow-up and surveillance.
